# Investigating the Functional Utility of the Left Parietal ERP Old/New Effect: Brain Activity Predicts within But Not between Participant Variance in Episodic Recollection

**DOI:** 10.3389/fnhum.2017.00580

**Published:** 2017-12-05

**Authors:** Catherine A. MacLeod, David I. Donaldson

**Affiliations:** ^1^Dementia Services Development Centre Wales, School of Healthcare Sciences, Bangor University, Bangor, United Kingdom; ^2^Psychological Imaging Laboratory, Psychology, Faculty of Natural Sciences, University of Stirling, Stirling, United Kingdom

**Keywords:** episodic memory, event-related potential (ERP), individual differences, left parietal effect, recollection

## Abstract

A success story within neuroimaging has been the discovery of distinct neural correlates of episodic retrieval, providing insight into the processes that support memory for past life events. Here we focus on one commonly reported neural correlate, the left parietal old/new effect, a positive going modulation seen in event-related potential (ERP) data that is widely considered to index episodic recollection. Substantial evidence links changes in the size of the left parietal effect to changes in remembering, but the precise functional utility of the effect remains unclear. Here, using forced choice recognition of verbal stimuli, we present a novel population level test of the hypothesis that the magnitude of the left parietal effect correlates with memory performance. We recorded ERPs during old/new recognition, source accuracy and Remember/Know/Guess tasks in two large samples of healthy young adults, and successfully replicated existing within participant modulations of the magnitude of the left parietal effect with recollection. Critically, however, both datasets also show that across participants the magnitude of the left parietal effect does not correlate with behavioral measures of memory – including both subjective and objective estimates of recollection. We conclude that in these tasks, and across this healthy young adult population, the generators of the left parietal ERP effect do not index performance as expected. Taken together, these novel findings provide important constraints on the functional interpretation of the left parietal effect, suggesting that between group differences in the magnitude of old/new effects cannot always safely be used to infer differences in recollection.

## Introduction

Since the 1980’s (Johnson, 1995) event-related potential (ERP) studies of episodic memory have routinely revealed parietal ‘retrieval success’ effects – neural activity associated with remembering events from the past. Specifically, ERPs to correctly recognized old stimuli exhibit greater positivity than ERPs to correctly rejected new stimuli, a difference that onsets around 500 ms post-stimuli, lasts for 200–400 ms, and is maximal over left parietal scalp electrodes. Given the scalp distribution and eliciting conditions, this ERP signal has become widely known as the ‘left parietal ERP old/new effect’ (Wilding et al., 2005; although other terms are used, e.g., the Late Positive Complex, see Friedman and Johnson, 2000). Over time the left parietal effect has become widely accepted as a robust neural correlate of memory retrieval, supporting dual process models of recognition memory (cf. Friedman and Johnson, 2000; Donaldson et al., 2003; Rugg and Curran, 2007) and being used as a neural marker of remembering in a wide variety of contexts.

The left parietal old/new effect has been examined using a wide variety of memory tasks and manipulations. Across many studies, it is clear that the magnitude of the left parietal effect increases in size with manipulations that increase levels of recollection (i.e., the conscious recovery of contextual information from prior study episodes). Notably, the left parietal effect has been shown to modulate with the Remember/Know (R/K) procedure (Smith, 1993; Düzel et al., 1997), depth of processing (Rugg et al., 1998), amount of information recollected (Vilberg et al., 2006; Vilberg and Rugg, 2009), accuracy and number of source judgments made (Wilding and Rugg, 1996; Trott et al., 1997), retrieval of associative information (Donaldson and Rugg, 1998), and number of repeated study-test blocks (Johnson et al., 1998). A seminal example is provided by Rugg et al. (1998), who used a within participant design to compare old/new effects elicited by retrieval of words that had originally received deep or shallow processing at study. In this case, participants exhibited better performance, and larger left parietal old/new effects, following deep (semantic) than shallow (perceptual) encoding. Taken together, the accumulated ERP data strongly suggests that increases in recollection lead to increases in left parietal old/new effect magnitude.

Studies where comparisons have been carried out between groups provide mixed evidence of the relationship between left parietal effect magnitude and recollection. For example, Van Petten et al. (2002) reported a posteriorly distributed ERP old/new effect recorded during an item recognition test that was modulated by the degree of stimulus ‘oldness.’ Contrary to expectations, however, ERP effect magnitude was not sensitive to whether participants were good or bad at the memory task. In contrast, Curran and Cleary (2003) found that performers with good recollection ability showed larger parietal effects for studied than similar items, whereas poor performers showed no significant ERP differences between studied and similar items. Importantly, however, even the poor performers (with no studied/similar ERP effects) could discriminate studied from similar lures behaviorally, putatively based on the differential engagement of recollection. Arguably, the variation in memory-related changes in the size of parietal effects in these two studies could reflect false negative/positive results due to a lack of power (comparisons between sub-groups of 12 participants for Van Petten et al., 2002, and sixteen for Curran and Cleary, 2003), or the use of different types of stimuli with Van Petten et al. (2002) using words, and Curran and Cleary using pictures (for evidence that ERP old/new effects are material specific see Yick and Wilding, 2008; MacKenzie and Donaldson, 2009; Galli and Otten, 2011). Similar concerns also apply to the findings of Schiltz et al. (2006), who failed to find significant correlations between behavioral measures of face recognition and left parietal effect magnitudes measured in young (*n* = 13) and older (*n* = 20) adults. Given concerns about the interpretation of null findings in the existing literature, one aim of the current study was to examine the relationship between behavioral and neural measures at a population level.

Overall the existing published studies broadly support the claim that the left parietal old/new effect is modulated by manipulations of recollection – typically characterized in terms of changes in the degree to which recollection is engaged, changes in the amount of information retrieved, or changes in the frequency of recollection. It is important to note that this characterization of the neural signal does not imply that recollection is a continuous rather than thresholded process *per se* (for further discussion of the distinction between behavioral and neural accounts of recollection see Murray et al., 2015). Rather, taken together, these findings have led to a consensus view that the left parietal old/new effect indexes episodic recollection (Wilding, 2000). Although often implicit within the literature, explicit claims to this effect are not uncommon, for example Johnson et al. (1998, p. 85) state that there is “a left parietal subcomponent, the magnitude of which is related to the participants ability to recollect.” Curran (2000, p. 933) notes: “A clear correlation has been established between recollection and the parietal old/new effect, but a better specification of the underlying cognitive processes is only beginning to emerge.” Similarly, Kipp et al. (2015, p. 380) argue that “A later old/new effect at parietal recordings, referred to as the parietal old/new effect, can be taken as the correlate of recollection” and Palombo et al. (2015, p. 107) state that “a late positive ongoing event-related brain potential (ERP) localized over parietal scalp areas that is a reliable neural marker of episodic recollection in association with correctly recognized stimuli.” In short, within the literature there is clear agreement that the left parietal effect is a reliable neural correlate of recollection – increases in the size of the left parietal old/new effect reflect increases in recollection (as reflected by changes in behavioral measures of memory).

Before introducing the current study we first clarify why it matters if a neural signal indexes a cognitive process. The majority of ERP findings described in the literature reflect within-participant (or intra- individual) modulations, whereby the magnitude of the old/new effect is sensitive to manipulations designed to influence recollection. Whilst typically only involving a limited number of conditions that preclude formal correlation analyses (e.g., Source Correct vs. Source Incorrect; retrieval of 1 vs. 2 items, etc.) such data nonetheless imply a correlation between behavior and neural activity, licensing the term ‘index.’ In this context, researchers can use the size of the left parietal old/new effect to infer changes in memory performance, and vice versa. Here we outline two scenarios that illustrate the kind of inferences that follow. Most straightforwardly, finding that the left parietal effect is smaller when participants use one encoding task (or material type, or retrieval strategy) rather than another supports the conclusion that the tasks (or materials, or strategies, etc.) differentially engage recollection. By extension, the observation of equivalent left parietal effects between participants with and without a neurological disorder (or personality type or gender, etc.) supports the view that recollection is equally engaged in each group. Importantly, these inferences do not rely on the specific nature of the variability between the neural and behavioral measures (i.e., whether it is linear or non-linear), simply that there is a systematic relationship.

The description of the left parietal old/new effect as a correlate or index of recollection clearly matters from the perspective of understanding and characterizing its functional role in memory. More importantly, perhaps, it also has significant consequences for the way in which ERP data can be used. For example, the fact that there is a relationship between neural and behavioral measures licenses the use of electrophysiological data in contexts that go beyond within participant manipulations of memory, including between-group comparisons such as good vs. poor performers (Curran et al., 2001; Van Petten et al., 2002; Wolk et al., 2009; Dockree et al., 2015), old vs. young participants (Li et al., 2004; Wolk et al., 2009; Dockree et al., 2015), men vs. women (Guillem and Mograss, 2005; Guillem et al., 2009), or participants exhibiting more or less false memories (Nessler et al., 2001; Morcom, 2015). Equally, because it indexes episodic recollection, the ERP effect is considered to be useful as a bio-marker of memory decline with disease (Olichney et al., 2008; Addante et al., 2012), memory deficiency in healthy adults (Palombo et al., 2015), and as a tool for assessing differences in the development of memory processes (Kipp et al., 2015). Whilst these scenarios vary in the nature of the comparisons being made, to produce interpretable results they all rely on comparisons of the magnitude of effects between participants, an approach that is licensed by the presence of a reliable relationship between behavioral and neural measures of recollection. The current paper examines whether this kind of comparison is valid.

## Experiment 1

In Experiment 1 we directly test the hypothesis that the magnitude of the left parietal old/new effect correlates with memory performance across participants. Given the general characterization of the role of the parietal effect in memory, and the evidence described above, we expected to find a direct relationship between patterns of memory related ERP activity and behavior. To be clear, we predicted that, across participants, the magnitude of the left parietal effect would increase in concert with changes in memory performance – better memory being associated with larger old/new effects. We tested this prediction using a larger than normal sample of participants (*n* = 122, providing excellent power), all of whom performed a standard item recognition memory test, discriminating between studied and unstudied words. To our surprise, whilst we replicated the expected pattern of memory related changes in the size of the left parietal effect within participants, we found no evidence for a relationship between the size of the left parietal effect and memory performance between participants.

### Methods

#### Participants

This work was carried out in accordance with the recommendations of The British Psychological Society ethical guidelines (The British Psychological Society, 2010). The University of Stirling Department of Psychology Ethics Committee gave ethical approval for Experiment 1, and participants were recruited from the University of Stirling. One hundred twenty-nine people (91 women; mean age = 20 years; age range = 17–35 years) participated and were reimbursed for participation at a rate of $5 per hour, or received course credit. All participants reported they were right-handed; native English speakers; with normal/corrected to normal vision; no history of color blindness, hearing difficulties, dyslexia, neurological problems, brain injury, CNS infection, drug or alcohol abuse, and had not or were not currently receiving treatment for a psychological illness. Saliva samples were taken for genotyping and participants completed a battery of cognitive, neuropsychological and psychometric assessments, of which only a proportion is reported here (for details of additional assessments see MacLeod, 2011; for genetic analysis results see MacLeod and Donaldson, 2014). In accordance with the Declaration of Helsinki, written informed consent from all participants was obtained prior to participation, and participants were fully debriefed at the end.

#### Stimulus Material

Stimuli were drawn from the MRC Psycholinguistic database (Coltheart, 1981), randomly selecting 148 six-letter words with a Kücera-Francis written frequency of 10–13 per million (Kučera and Francis, 1967). This list was hand-filtered to remove unsuitable words such as identifiers, emotive/arousing words, and visually similar words. Forty words were removed during hand filtering leaving 108, of which eight were used during practice trials and 100 in experimental trials. The 100 experimental words were alphabetized and divided into two lists, to be used as studied and unstudied words, by alternately assigning words to each (mean Kücera-Francis written frequency for list *A* = 11.16, for list *B* = 11.24). List presentation was counterbalanced across participants.

#### Experimental Procedure

The word recognition memory task was part of a battery of four single item recognition tasks (differing by stimulus type), with task order counterbalanced across participants. The task consisted of a short encoding phase (50 words) during which participants were instructed to try and remember each word for a later memory test. Words were presented electronically using E-Prime 1.1 software (Psychology Software Tools Inc., 2008) for 1000 ms each, and were preceded by a cross-hair presented for 2000 ms. A 1-min break between study and test was included to reduce reliance on working memory, during which participants were instructed to relax and rest their eyes. At test participants were presented with 100 words (50 old/50 new) responding ‘old’ or ‘new’ as quickly and accurately as possible. Responses were made with left/right index fingers on buttons ‘1’ or ‘5’ (counterbalanced across participants) of a five-button response box. Stimuli were presented for 1000 ms each during the test phase and were followed by an infinite blank screen, with responses triggering the next trial. A cross-hair presented for 2000 ms preceded each item and indicated the start of a new trial. Words were presented in white, bold, 18 point Courier New font in the center of the screen against a black background.

#### Electroencephalogram Acquisition

Scalp voltages were recorded from 62 silver/silver chloride electrodes, mounted in an elastic cap (QuickCap) using an extended version of Jasper (1958) international 10/20 system, connected to a Neuroscan amplifier (SynAmps^2^) and recorded with Neuroscan software Aquire/Edit 4.3/4.4 (Neuromedical Supplies^1^). Impedances were kept below 5 KΩ and data was digitized at a rate of 250 Hz, sampling at 4 ms/point, and a band-pass filter of 0.1–40 Hz was used to attenuate both high and low frequencies. Signals were amplified with a gain of 2010.

#### Data Analysis

##### Behavioral data analysis

Behavioral and ERP data were filtered for markedly quick or slow response times, excluding trials below 300 ms and above twice the mean response time for each condition. Memory accuracy was estimated using a two-high threshold model (Snodgrass and Corwin, 1988). The discrimination index (*Pr = pHit - pFA*) was used to correct scores for guesses, and the bias index (*Br = pFA/[1-Pr]*) was used to estimate probability of guessing ‘old’ when uncertain. Although *Pr* and *Br* are standard measures they are inherently vulnerable; in cases where participants make no errors in their recognition judgments *Pr* would be equal to 1, resulting in the division of the proportion of False Alarms by 0 in the calculation of *Br*. Inline with Snodgrass and Corwin (1988), all Hit and False Alarm rates were adjusted to correct for this mathematical impossibility so that Hit rate equals (*number of Hits*+*0.5*)/(*the number of old stimuli*+*1*), and False Alarm rate equals (*number of False Alarms*+*0.5*)/(*the number of new stimuli +1)*. Data from three participants were excluded from analysis due to low discrimination (*Pr* < 0.2), ensuring neural activity was examined only for participants who were unquestionably remembering.

Bayes Factor analyses were carried out for all correlational analyses to compute the probability of the observed data under the null and alternative hypotheses, calculating a weighted average likelihood ratio – the Bayes Factor (BF_10_). Values greater than 1 indicate a greater likelihood of the data occurring under the alternative hypothesis, with values less than 1 under the null hypothesis (Wetzels and Wagenmakers, 2012). Jeffreys (1961) proposed a category scheme to indicate the evidential impact of different Bayes Factors, which was adapted by Wetzels and Wagenmakers (2012). On this scale a BF_10_ of 1 indicates “no evidence,” with scores of less than 3 (for the alternative hypothesis) or more than 1/3 (for the null hypothesis) considered inconclusive or “anecdotal evidence” (Wetzels and Wagenmakers, 2012, p. 1060). Values of 1/10 – 1/3 are considered “substantial evidence” continuing up to <1/100 at the top of the scale, which is “decisive evidence” for the null hypothesis. Conversely scores of 3–10 indicate “substantial evidence” continuing up until >100 for “decisive evidence” in favor of the alternative hypothesis (for the full scale see Wetzels and Wagenmakers, 2012, p. 1060, **Table 1**). Bayes Factor analyses were conducted using JASP software version 0.7.1.12 (Love et al., 2015), with all other analyses carried out using IBM SPSS software version 22.

**Table 1:** Experiment 1 old/new recognition task behavioral results for (A) all participants; and (B) high and low performance groups.

	Group	Hit rate (%)	False alarm rate (%)	*Pr*	*Br*	Hit RT (ms)	CR RT (ms)
(A)	All (*n* = 122)	73 (12)	18 (11)	0.54 (0.17)	0.39 (0.16)	823 (138)	898 (157)
(B)	High *Pr***** (*n* = 24)	87 (8)	6 (4)	0.79 (0.09)	0.36 (0.15)	822 (176)	895 (181)
	Low *Pr***** (*n* = 24)	62 (8)	21 (10)	0.40 (0.09)	0.36 (0.14)	853 (174)	916 (194)


*Mean scores with standard deviation given in brackets.*

##### Event-related potentials

Electroencephalogram (EEG) data was re-referenced off-line to linked-mastoids. Epochs of 2100 ms were extracted from EEG, time-locked to stimulus onset (0 ms), with a 100 ms pre-stimulus baseline. Eye-blinks were removed using the ocular artifact reduction procedure (OAR) in Neuroscan Edit software (version 4.3). The OAR uses an eye movement correction algorithm. First, a set of eye-blink potentials were identified from the vertical electrooculogram (EOG) channels. Blinks were selected manually from the EEG to ensure that only representative blinks were included, with a minimum threshold of 32 blinks per participant. Second, these blink trials were used to generate an average blink artifact for each participant, allowing the computation of blink transmission coefficients for each channel. Third, these coefficients were used to subtract the contribution of blink artifacts from the raw EEG waveforms on a point by point basis. Following OAR correction trials where drift was greater than ±75 μV or where the signal exceeded ±100 μV on any electrode were excluded. Data was smoothed using a rolling 5-point average window. ERPs were based on a minimum of 16 artifact-free trials per condition, per participant, with averages formed to Hit and Correct Rejection (CR) responses for each participant. Data from four participants were excluded due to an insufficient number of artifact-free trials (<16) in any response category, with 122 participants included in the analysis.

### Results and Discussion

#### Does the Left Parietal Old/New Effect Correlate with Performance?

##### Behavioral results

Behavioral data is presented in **Table 1**, row A. Participants were successfully able to complete the word recognition task with mean discrimination accuracy above chance [t(121) = 34.53, *p* < 0.001]. Overall participants had a conservative decision bias. Mean response times (RT) for Hits were significantly [t(121) = 9.83, *p* < 0.001] faster than CR responses.

##### ERP results

**FIGURE 1:**
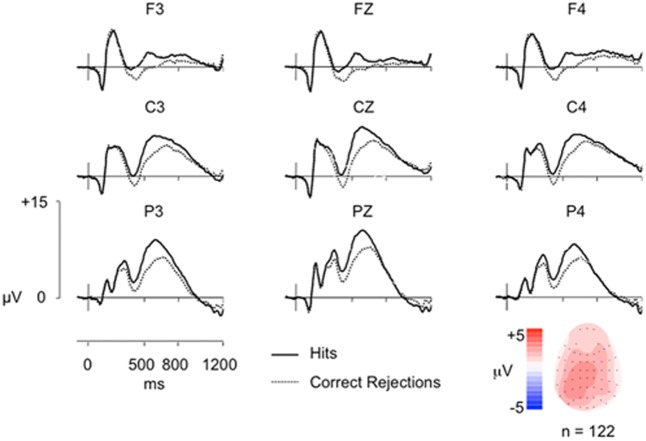
Grand-average ERP old/new effects from a simple word recognition task (*n* = 122) at representative Frontal (F), Central (C), and Parietal (P) electrodes, along with a topographic map illustrating the old/new effect distribution from 500 to 800 ms. The vertical scale indicates electrode amplitude (microvolts) and the horizontal scale indicates change in time (milliseconds). The color scale indicates Hit-CR difference size (microvolts). Hit ERPs are more positive going than CRs from ∼300 ms post-stimulus onset (0 ms), reconverging by epoch end, with a maximal difference over left parietal electrodes (P3).

Average ERPs were formed for each participant, with the mean number of trials for Hit and CR conditions 29 and 32, respectively (minimum = 16/44, maximum = 19/45, mean trials rejected = 16%/19%). ERP data are shown in **Figure 1**, indicating a clear left parietal old/new effect during the 500–800 ms window. These data were analyzed using ANOVA (Greenhouse-Geisser corrected for non-sphericity as appropriate), with factors of condition (Hits/CRs), location (frontal/fronto-central/central/centro-parietal/parietal), hemisphere (left/right), and electrode site (inferior/medial/superior); including electrodes at sites 5/3/1 on the left and 6/4/2 on the right, at each location F/FC/C/CP/P. Analysis of data from the 500–800 ms time-window revealed a significant main effect of condition [*F*(1,121) = 42.55, *p* < 0.001], and significant interactions between condition and site [*F*(1,129) = 21.86, *p* < 0.001]; condition, location and hemisphere [*F*(1,160) = 6.87, *p* = 0.005]; condition, location and site [*F*(2,266) = 6.69, *p* = 0.001]; and condition, location, hemisphere and site [*F*(3,395) = 10.16, *p* < 0.001]. No significant interactions between condition and location [*F*(1,144) = 2.16, *p* = 0.14]; condition and hemisphere [*F*(1,121) = 3.24, *p* = 0.074]; or condition, hemisphere and site [*F*(1,151) = 1.34, *p* = 0.256] were found. As expected, ERPs to Hits are more positive going than those to CRs, a difference clearly maximal over the left hemisphere at centro-parietal and parietal electrodes (and largest at medial electrodes).

**FIGURE 2:**
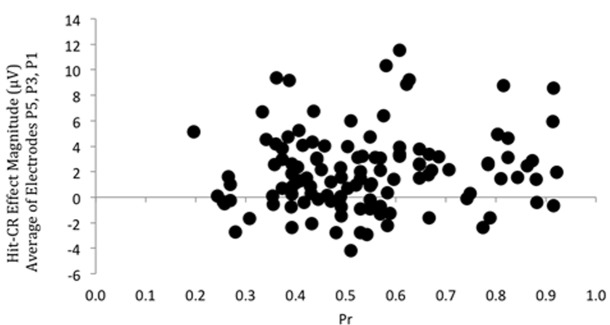
Scatterplot depicting the relationship between *Pr* and ERP Hit/CR effect magnitude in microvolts, from 500 to 800 ms at left parietal electrodes (averaged across P5, P3, P1). No significant correlation was found.

Having demonstrated that a left parietal old/new effect was present, we then examined the relationship between episodic memory performance and the underlying neural signal: a correlation was performed on discrimination accuracy (*Pr*) scores and left parietal effect magnitude, as indexed by the mean old/new difference between 500 and 800 ms. Due to possible variation in maximal old/new difference site across participants an average left parietal old/new effect magnitude was calculated by averaging across electrodes P5, P3, and P1. These data are shown in **Figure 2**; covering a substantial range of scores in both performance (*Pr* values from 0.20 to 0.92) and neural activity (voltage values from -4.19 to 11.52 μV). Importantly, despite the degree of variability and large sample size involved, the correlation was not significant (*r* = 0.133, *p* = 0.144): there is no systematic relationship between the behavioral and neural measures of memory across participants. Furthermore, Bayes Factor analyses yields a value of BF_10_ = 0.324, suggesting “substantial evidence” for the null hypotheses (*H*_0_), according to the adapted Jeffreys (1961) category scheme in Wetzels and Wagenmakers (2012). In addition, no significant correlations were found between left parietal effect magnitude response bias (*Br*) (*r* = 0.032, *p* = 0.725, BF_10_ = 0.120) and Bayes Factor values suggest “substantial evidence for *H*_0_”. A sensitivity analysis of a bivariate normal model correlation using G^∗^power 3.1 software (Faul et al., 2009) with a two tailed alpha value of 0.05 showed that a sample size of 122 with a power of 0.8 could detect a small to medium effect size (Cohen, 1988) of 0.250, with a correlation interval of ±0.178.

#### Comparing High and Low Performers

Given the unexpected results of a lack of correlation between *Pr* and left parietal effect magnitude across participants, we carried out additional analyses to further examine the relationship between behavioral and neural measures. First, given the range of behavioral performance within the dataset, we examined two subsets of participants that allowed us to directly compare high and low performers (on the basis of differences in *Pr*), whilst strictly controlling for differences in bias and reaction time. Participants with *Pr* ≥ 0.65 were classified as high performers and those with *Pr* ≤ 0.55 (but greater than *Pr* = 0.2) were classified as low performers, excluding mid-range performers. Two performance groups were created, selecting 24 participants in each group, matched for mean and standard deviation decision bias (*Br*) scores, as well as keeping mean Hit and CR response times as consistent as possible. The behavioral characteristics of the high and low performance groups are presented in **Table 1**, row B. To confirm that the ERP analyses are comparing groups that differ by performance, and not other behavioral measure, independent samples *t*-tests were conducted. The results confirm that the two groups were significantly different in discrimination accuracy [*t*(46) = -15.31, *p* < 0.001], but did not differ in bias [*t*(46) = -0.02, *p* = 0.985], nor Hit [*t*(46) = 0.6, *p* = 0.551], or CR [*t*(46) = 0.38, *p* = 0.703] response times.

##### ERP results

**FIGURE 3:**
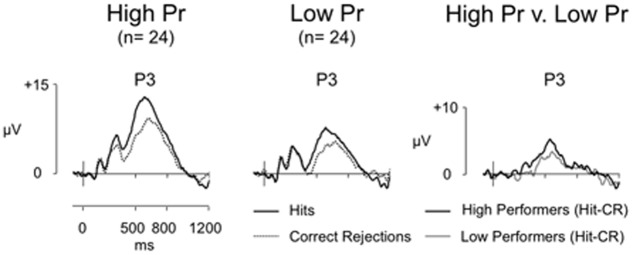
Grand-average ERP old/new effects for high (*n* = 24) and low (*n* = 24) performance groups, and difference (Hit-CR) waveforms for each group at electrode P3 (scales as per **Figure 1**). For both groups Hit ERPs are more positive going than CRs, however, the difference in old/new effect magnitude at electrode P3 between high and low performers is minimal.

Average ERPs were formed for each participant, with grand averages formed for each group, (high *Pr* group trials: Hit/CR mean = 34/37, respectively, minimum = 27/26 maximum = 43/43, mean trials rejected = 18%/18%; low *Pr* group trials: Hit/CR mean = 25/32, respectively, minimum = 17/22, maximum = 39/44, mean trials rejected = 13%/15%). **Figure 3** shows grand average ERPs for Hit and CR responses at electrode P3 for high and low performers. Clear old/new effects can be seen for both performance groups. Although both groups clearly elicited significant old/new effects, the key question is whether better recognition performance is associated with a significant increase in left parietal effect magnitude. To answer this question we directly compared the old/new effects for high and low performers using difference waveforms. Analysis focused initially on left parietal effect magnitude (average of electrodes P5, P3, P1), using a planned *t*-test this revealed no significant difference in the size of the left parietal effect [*t*(46) = -0.84, *p* = 0.405] – despite the large difference in *Pr*. Additional analyses were conducted using ANOVA, with between participants factor of group (high *Pr*/low *Pr*), and within participant factors of location (F/FC/C/CP/P), hemisphere (L/R) and site (I/M/S). This analysis also revealed no significant main effect of *Pr* group [*F*(1,46) = 0.709, *p* = 0.404] or interactions involving *Pr* group {location and *Pr* [*F*(1,59) = 0.023, *p* = 0.925]; hemisphere and *Pr* [*F*(1,46) = 3.672, *p* = 0.062]; site and *Pr* [*F*(1,48) = 0.304, *p* = 0.593]; location, hemisphere, and *Pr* [*F*(1,53) = 2.831, *p* = 0.093]; location, site, and *Pr*; [*F*(3,118) = 0.078, *p* = 0.957]; hemisphere, site, and *Pr* [*F*(1,56) = 1.402, *p* = 0.248]; location, hemisphere, site, and *Pr* [*F*(3,117) = 1.325, *p* = 0.271]}, confirming that neither the magnitude nor distribution of the old/new effects differed reliably across groups. The lack of significant differences between groups is striking and reinforces the correlational result; even when differences in reaction time and bias are taken into account, the left parietal effect is not modulated by recognition performance.

### Summary

The present experiment aimed to investigate the relationship between left parietal old/new effect magnitude and recognition accuracy: contrary to expectation we found no significant correlation between neural and behavioral measures of episodic memory across participants. These findings stand in stark contrast to our predictions and question the putative relationship between the left parietal effect and behavioral measures of episodic memory retrieval in a between participant context. In short, our data suggest that, across participants, the left parietal effect associated with memory retrieval is not a good predictor of (i.e., robust neural correlate of) memory performance.

Our results question the circumstances under which the left parietal old/new ERP effect can be viewed as an ‘index’ of episodic recollection. As expected, across all participants, our initial analysis did reveal the ‘typical’ left parietal effect seen in word recognition studies: Hits generated more activity than CRs over left parietal electrodes between 500 and 800 ms. Importantly, however, analysis directly comparing the magnitude of the left parietal effect (averaged over electrodes P5, P3, and P1) and memory performance (either Hit rate or discrimination) failed to find a significant correlation. Moreover, comparison of two groups of participants selected to represent high and low performance also failed to find significant differences in the magnitude or distribution of ERP effects.

Although the present results are surprising, our use of a simple old/new recognition task prohibits an assessment of whether memory performance was based on recollection (rather than, for example, familiarity or unconscious priming). We chose a simple recognition task on the basis that this is the most typical experimental paradigm used in studies of episodic memory – and that performance on this task is well known to reliably engage recollection – fully expecting to find a significant correlation. Nonetheless, in the absence of an explicit measure of recollection we are unable to definitively rule out the possibility that participants may have based their performance on non-recollective memory in this instance, or a combination of recollection and familiarity that weakened the potential to detect a significant relationship. If this were the case, then variability in overall memory performance may not have correlated with left parietal effect magnitude simply because differences in memory were not driven by recollection *per se*. Consequently, before considering the implications of our findings further, we first present a follow up experiment that addresses these concerns.

## Experiment 2

Having unexpectedly found that neural signals putatively associated with recollection do not correlate with memory performance on a standard old/new recognition task, we designed a follow up experiment that explicitly measures recollection. In practice, of course, no single experimental method provides an unequivocal assessment of recollection – all available methods can be criticized to some degree (see Yonelinas, 2002). We therefore employed two distinct approaches. To provide an objective measure of criterial recollection, we employed a source memory (SM) task (Wilding and Rugg, 1996), requiring participants to study words in two colors, and report the color as a source attribute at test (Cruse and Wilding, 2009). In addition, to provide a subjective measure of recollection, that will capture a broader array of recollected experiences, we separately employed an item recognition task along with the Remember/Know procedure (Tulving, 1985; Gardiner et al., 1998, although for criticism see Wais et al., 2008), asking participants to indicate whether each retrieval experience was associated with recollection (‘Remember’), familiarity (‘Know’) or guessing. Across these two tasks we can be confident that where variability in memory performance tracks with source accuracy rates or ‘Remember’ rates, participants are indeed recollecting. To be clear, if the left parietal effect provides a universal index of recollection two simultaneous findings should result. First, across participants, the magnitude of the left parietal effect will increase in concert with changes in recollection – more recollection being associated with larger old/new effects. Second, within participants, the magnitude of the left parietal effect will be larger in conditions associated with recollection (i.e., ‘Remember’ and Source Correct, compared to ‘Know’ and Source Incorrect). These data therefore provide a further opportunity to investigate the relationship between parietal memory signals and episodic retrieval – and potentially to replicate the unexpected discovery that across participants the left parietal old/new effect does not correlate with episodic memory.

### Methods

Experiment 2 included two separate episodic memory tasks: a SM task and a single item word recognition task with secondary Remember/Know/Guess (RKG) judgment, completed one week later. EEG acquisition and analysis were as described for Experiment 1. The University of Stirling Department of Psychology Ethics Committee granted ethical approval for Experiment 2, with participants recruited from the University of Stirling. Sixty-six participants completed the SM task (33 women, mean age = 20 years, age range 18–28 years, with inclusion/exclusion criteria as stated for Experiment 1), with 63 participants also completing the RKG task (three participants failed to return for the second session). Twelve participants from Experiment 1 also participated in Experiment 2. Participants were reimbursed for participation at a rate of $7.50 per hour, or received course credit. Written informed consent from all participants was obtained prior to participation, and participants were fully debriefed at the end.

The SM task asked participants to remember words (a set of 5–7 letter words equivalent to those used in Experiment 1) presented during a study phase and the color they were presented in (pink/yellow, as utilized by Cruse and Wilding, 2009). Participants were presented with seven study-test blocks of 24 study words (12 pink/12 yellow) and 48 test words in each block. Words were presented for 1000 ms at study and test, and were preceded by a cross-hair presented for 2000 ms. During the test phase participants were asked to indicate if the word (presented in white) had been presented at study (‘old’ or ‘new’). An infinite blank screen followed test word presentation to allow participants time to respond, with responses triggering the next trial. If an ‘old’ response was made, participants were asked to indicate what color the word was presented in at study (‘pink’ or ‘yellow’) with responses triggering the next trial. Old/new judgments were made with left/right index fingers on buttons ‘1’ or ‘5,’ and pink/yellow responses on buttons ‘2’ or ‘4’ (counterbalanced across participants). There was a 1-min break between study and test phases, and a participant led break between blocks.

The RKG task replicated the old/new recognition task presented in Experiment 1 (using a new set of 5–7 letter words) with an additional secondary Remember/Know/Guess judgment at test following ‘old’ judgments. Participants were presented with two study-test blocks of 50 ‘old’ and 50 ‘new’ words in each block. As per Experiment 1 each word was presented for 1000 ms and was preceded by a cross-hair presented for 2000 ms. At test an infinite blank screen followed word presentation, with responses triggering the next trial. If participants made an ‘old’ response a secondary R/K/G response was required, during which participants indicated if they specifically remembered seeing the word in the study phase, if they just knew that it had been presented, or if they were guessing. Responses again triggered the next trial. There was a 1-min break between study and test phases, and a participant led break between blocks. Old/new judgments were made with left/right index fingers on buttons ‘1’ or ‘5,’ with ‘Remember’ and ‘Know’ responses on buttons ‘2’ or ‘4’ (counterbalanced across participants), and ‘Guess’ responses always made with the center button (‘3’). Responses triggered the next trial.

### Results and Discussion

#### An Objective Measure of Recollection – Source Memory

Sixty-one participants were included in the SM task analysis [excluding one participant due to low discrimination accuracy (*Pr* < 0.2) and four participants due to an insufficient number of artifact-free trials (2 < 16 HSC trials, 2 < 16 HSI trials)]. Behavioral data are presented in **Table 2**, row A. Participants were successfully able to complete the SM task with mean discrimination accuracy significantly above chance [*t*(60) = 28.99, *p* < 0.001]. Overall participants exhibited a conservative decision bias, and had a source judgment accuracy of 64% (*SD* = 12%). There was no significant difference between the Hit and CR response times [*t*(60) = -0.1, *p* = 0.922].

**Table 2:** Experiment 2 behavioral results for (A) Source Memory (SM) task all participants; (B) Remember/Know/Guess (RKG) task all participants; (C) RKG task larger subgroup without ‘Know’ response analysis.

	Group	Hit rate (%)	False alarm rate (%)	*Pr*	*Br*	Hit RT (ms)	CR RT (ms)
(A)	SM (*n* = 61)	71 (14)	13 (11)	0.57 (0.15)	0.31 (0.22)	1179 (285)	1185 (525)
(B)	RKG (*n* = 20)	71 (10)	19 (12)	0.52 (0.14)	0.38 (0.17)	1212 (198)	1225 (243)
(C)	RKG (*n* = 53)	72 (17)	18 (14)	0.54 (0.19)	0.39 (0.24)	1150 (239)	1255 (460)


*Mean scores with standard deviation given in brackets.*

**FIGURE 4:**
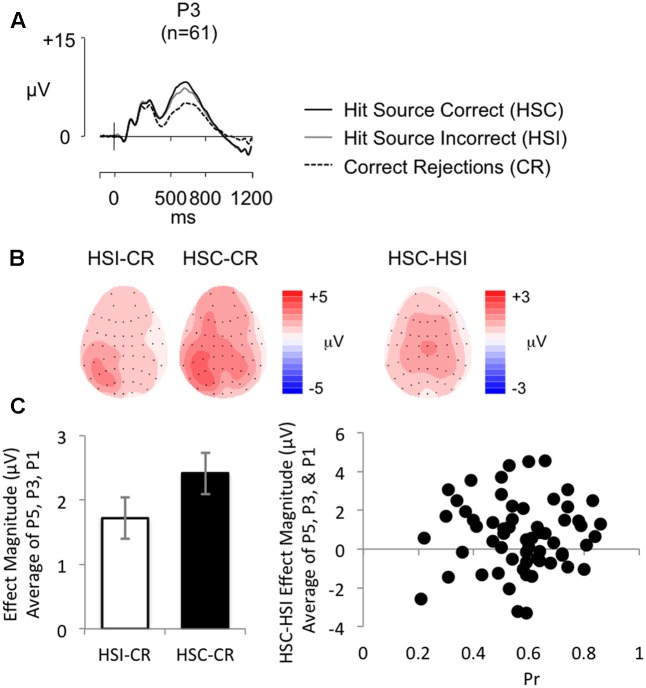
**(A)** Grand-average ERP old/new effects from a word-color source task (*n* = 61), at electrode P3, for Hit Source Correct (HSC), Hit Source Incorrect (HSI), and CR responses (scales as per **Figure 1**). **(B)** Topographic maps illustrate clear left parietal effects from 500 to 800 ms for HSI and HSC responses compared to CR responses, and a more centrally distributed difference for HSC compared to HSI responses. **(C)** Bar graph (left) illustrating mean left parietal old/new effect magnitude (average of electrodes P5, P3, P1) for HSI (white bars) and HSC (black bars) responses, and a scatterplot (right) depicting the relationship between *Pr* and ERP HSC/HSI effect magnitude in microvolts, from 500 to 800 ms at left parietal electrodes (averaged across P5, P3, P1). No significant correlation was found.

**Figure 4A** shows grand average ERPs for the SM task at electrode P3 for Hit trials where the source was correctly remembered (HSC), Hit trials where the source was not correctly remembered (HSI) and CR responses (mean number of HSC/HSI/CR trials = 64/35/114; minimum trials = 30/16/74; maximum = 108/88/143; mean percentage of trials rejected = 17%/16%/21%). Topographic maps (**Figure 4B**) show old/new effect distributions between 500 and 800 ms post-stimulus. As expected both HSC and HSI responses are more positive going than CRs, with the difference appearing maximal over left parietal electrodes. The HSC-CR difference appears slightly more widespread in distribution; nonetheless the difference is once again clearly maximal over left posterior electrodes. HSC responses are more positive than HSI responses, however, this difference appears to have a central maximum. As per Experiment 1, an average left parietal old/new effect was calculated (average of electrodes P5, P3, P1). As illustrated in **Figure 4C** (left), mean old/new effect magnitude across these electrodes were larger for HSC than HSI responses.

Analysis (employing equivalent ANOVA structure to that used in Experiment 1) comparing HSC and HSI responses are presented in **Table 3**, column 2. The results indicate that, overall, Hits were more positive going than CRs, a difference greatest at superior sites at central and centro-parietal locations. Further analysis (employing equivalent ANOVA structure to that used in Experiment 1) comparing HSC and CR responses (**Table 3**, column 3) indicates that compared to CRs, HSCs were more positive going, a difference greatest over left inferior and medial posterior electrodes. The old/new difference was more uniform in distribution across electrodes over anterior locations, and was larger at superior and medial electrodes over right posterior locations.

**Table 3:** Experiment 2 source memory task old/new effect ANOVA results.

SM	HSC vs. HSI (*n* = 61)	HSC vs. CRs (*n* = 61)
C	*F*(1,60) = 10.21, *p* = 0.002^∗^	*F*(1,60) = 31.87, *p* < 0.001^∗^
C×L	*F*(1,76) = 0.15, *p* = 0.754	*F*(1,80) = 3.44, *p* = 0.055
C×H	*F*(1,60) = 0.03, *p* = 0.871	*F*(1,60) = 10.83, *p* = 0.002^∗^
C×L×H	*F*(2,100) = 0.63, *p* = 0.507	*F*(1,85) = 4.34, *p* = 0.027^∗^
C×S	*F*(1,68) = 9.50, *p* = 0.002^∗^	*F*(1,65) = 4.75, *p* = 0.030^∗^
C×L×S	*F*(3,163) = 3.54, *p* = 0.019^∗^	*F*(2,130) = 12.02, *p* < 0.001^∗^
C×H×S	*F*(1,74) = 0.39, *p* = 0.579	*F*(1,86) = 9.81, *p* = 0.001^∗^
C×L×H×S	*F*(4,211) = 0.09, *p* = 0.978	*F*(3,193) = 12.16, *p* < 0.001^∗^


*Analyses included data from 30 electrodes, with factors of Condition (C), Location (L), Hemisphere (H), and Site (S). Significant results (*p* < 0.05) are highlighted by an asterisk (^∗^).*

Analysis of behavioral data revealed a significant positive correlation between accuracy of source judgment and old/new recognition performance (as indexed by *Pr*) [*r* = 0.298, *p* = 0.020]. This result was expected given the dependency of Source Correct judgments on prior Hit judgments, although Bayes Factor analysis (BF_10_ = 2.264) suggests only “anecdotal evidence” for the alternative hypothesis. Similarly, analysis of ERP data revealed a significant positive correlation between HSC/HSI effect magnitude and HSC/CR effect magnitude (*r* = 0.362, *p* = 0.004, BF_10_ = 8.891), although here Bayes Factor analysis indicates substantial evidence for the alternative hypotheses. Importantly, however, there were no significant correlations between ERP and behavioral data: more specifically, between HSC/HSI effect magnitude (average of electrodes P5, P3, P1) and *Pr* (*r* = 0.019, *p* = 0.884, BF_10_ = 0.161, see **Figure 4C** (right) for a scatterplot depicting the relationship between HSC/HSI effect magnitude and *Pr*), *Br* (*r* = 0.027, *p* = 0.839, BF_10_ = 0.163), or source judgment accuracy (*r* = 0.212, *p* = 0.102, BF_10_ = 0.589). Bayes Factors analyses showed “substantial evidence” for the null hypothesis, with the exception of the correlation between HSC/HSI and *Br*, which only indicated “anecdotal evidence.” There were also no significant correlations between HSC/CR effect magnitude (average of electrodes P5, P3, P1) and *Pr* (*r* = 0.120, *p* = 0.357, BF_10_ = 0.242), *Br* (*r* = -0.058, *p* = 0.657, BF_10_ = 0.176), or source judgment accuracy (*r* = 0.033, *p* = 0.802, BF_10_ = 0.165), with Bayes Factor for each condition indicating “substantial evidence” in support of the null hypothesis. A sensitivity analysis of a bivariate normal model correlation using G^∗^power 3.1 software (Faul et al., 2009) with a two tailed alpha value of 0.05 showed that a sample size of 61 with a power of 0.8 could detect a medium to large effect size (Cohen, 1988) of 0.349, with a correlation interval of ±0.252.

The results from the SM task analyses suggest that left parietal activity, whether measured by comparing Source Correct Hits with either Source Incorrect Hits or CRs, not only fails to track with variations in memory performance (*Pr*), but it also fails to track an explicit measure of recollection (SM accuracy) across participants. Whilst SM is a standard measure of recollection, and we have tried to isolate recollection by comparing Source Correct and Source Incorrect responses, one possible explanation is that people were able to make source judgments based on an absence of information (Leynes, 2012), or based on familiarity or guessing rather than recollecting the source *per se*. Furthermore, Leynes and Nagovsky (2016, p. 179) found that successful source judgments were not always accompanied by a left parietal old/new effect, differing as a function of encoding focus, which they suggest may be attributable to “recollection based on partial information.” Another possible explanation for the lack of correlation between neural and behavioral measures of memory is that parietal activity reflects the subjective experience of recollecting, rather than the actual recovery of information (Simons et al., 2010). Thus one explanation for the current findings is that participants in the SM task were able to correctly identify which color a word was presented in without experiencing a strong subjective sense of recollection. Based on these concerns, a second word recognition task was included in which participants were asked to indicate whether each retrieval experience was associated with a sense of recollection (‘Remembering’), familiarity (‘Knowing’) or that they were “Guessing.” The RKG paradigm provides an estimate of the subjective experience of recollection associated with successful retrieval.

#### A Subjective Measure of Recollection – Remember/Know/Guess

Only 20 participants met the inclusion criteria for analysis of both ‘Remember’ (R) and ‘Know’ (K) trials of the RKG task [excluding four participants due to low discrimination accuracy (*Pr* < 0.2) and 39 participants due to an insufficient number of artifact-free trials (6 < 16 R trials, 33 < 16 K trials)]. Behavioral scores are presented in **Table 2**, row B. Participants were successfully able to complete the task with mean discrimination accuracy above chance [*t*(19) = 16.33, *p* < 0.001]. Overall participants exhibited a conservative decision bias, and on average classified 51% (*SD* = 13%) of Hit trials as being ‘Remembered,’ 39% (*SD* = 12%) as ‘Known,’ and 9% (*SD* = 10%) as ‘Guesses.’ An adjusted ‘Remember’ score (percentage of Hit R – percentage of False Alarm R) was calculated to account for potential response bias [mean adjusted R = 39% (*SD* = 20%)]. ‘Guess’ responses were eliminated from any further analysis. There was no significant difference between the Hit and CR response times [*t*(19) = -0.26, 0.795].

**FIGURE 5:**
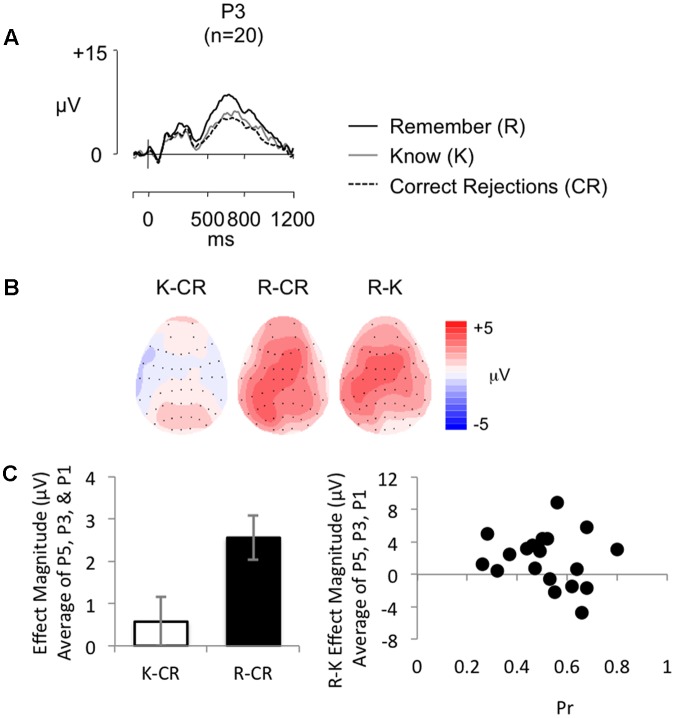
**(A)** Grand-average ERP old/new effects from a word recognition test with secondary RKG judgment (*n* = 20), at electrode P3, for ‘Remember’ (R), ‘Know’ (K), and CR responses (scales as per **Figure 1**). **(B)** Topographic maps illustrate the distribution of the old/new difference between 500 and 800 ms, highlighting the absence of a K-CR effect, and the presence of a left parietal effect for R responses. **(C)** Bar graph (left) illustrating mean left parietal old/new effect magnitude (average of electrodes P5, P3, P1) for K (white bars) and R (black bars) responses, and a scatterplot (right) depicting the relationship between *Pr* and ERP R/K effect magnitude in microvolts, from 500 to 800 ms at left parietal electrodes (averaged across P5, P3, P1). No significant correlation was found.

**Figure 5A** shows grand average ERPs for the RKG task at electrode P3 for R, K, and CR responses (mean number of R/K/CR trials = 31/23/63; minimum trials = 18/16/42; maximum = 55/42/79; mean percentage of trials rejected = 16%/15%/23%). Topographic maps (**Figure 5B**) show effect distributions between 500 and 800 ms post-stimulus. As expected R responses are more positive going than K responses and CR responses, with minimal difference evident between K and CR responses. The distribution of the R–K effect is central across central electrodes, becoming more left lateralized over parietal electrodes, with a similar pattern for the R–CR effect, which is more right lateralized over frontal electrodes. As illustrated by **Figure 5C** (left), mean old/new effect magnitude across electrodes P5, P3, and P1 was larger for R than K responses.

Analysis (employing equivalent ANOVA structure to that used in Experiment 1) comparing R and K responses are presented in **Table 4**, column 2. The results indicate that, overall, Hits were more positive going than CRs, a difference greatest at superior sites over central and centro-parietal locations, and more widespread across parietal locations. Further analysis (employing equivalent ANOVA structure to that used in Experiment 1) comparing R and CR responses (**Table 4**, column 3) shows the same pattern of results.

**Table 4:** Experiment 2 Remember/Know/Guess task old/new effect ANOVA results.

RKG	R vs. K (*n* = 20)	R vs. CRs (*n* = 20)	R vs. CRs (*n* = 53)
C	*F*(1,19) = 11.43, *p* = 0.003^∗^	*F*(1,19) = 13.22, *p* = 0.002^∗^	*F*(1,52) = 41.11, *p* < 0.001^∗^
C×L	*F*(1,21) = 0.47, *p* = 0.516	*F*(1,21) = 0.17, *p* = 0.712	*F*(1,60) = 9.48, *p* = 0.002^∗^
C×H	*F*(1,19) = 1.37, *p* = 0.257	*F*(1,19) = 0.89, *p* = 0.358	*F*(1,52) = 9.04, *p* = 0.004^∗^
C×L×H	*F*(1,29) = 1.31, *p* = 0.279	*F*(1,27) = 3.3, *p* = 0.067	*F*(1,74) = 7.04, *p* = 0.005^∗^
C×S	*F*(1,20) = 1.6, *p* = 0.222	*F*(1,20) = 4.11, *p* = 0.055	*F*(1,56) = 5.46, *p* = 0.021^∗^
C×L×S	*F*(2,46) = 6.54, *p* = 0.002^∗^	*F*(2,38) = 7.04, *p* = 0.003^∗^	*F*(2,112) = 9.71, *p* < 0.001^∗^
C×H×S	*F*(1,26) = 3.77, *p* = 0.051	*F*(2,30) = 2.1, *p* = 0.149	*F*(1,68) = 15.54, *p* < 0.001^∗^
C×L×H×S	*F*(4,68) = 0.28, *p* = 0.87	*F*(2,41) = 1.02, *p* = 0.373	*F*(3,145) = 6.54, *p* = 0.001^∗^


*Analyses included data from 30 electrodes, with factors of Condition (C), Location (L), Hemisphere (H) and Site (S). Significant results (*p* < 0.05) are highlighted by an asterisk (^∗^).*

The behavioral findings indicate that, on average, participants’ predominately experienced a sense of recollection when completing the task. No significant positive correlations were found between the proportion of ‘Remember’ responses and *Pr* (*r* = 0.012, *p* = 0.960), or with adjusted ‘Remember’ responses and *Pr* (*r* = 0.435, *p* = 0.055), with Bayes Factor analysis indicating “substantial evidence” for the null hypothesis (BF_10_ = 0.277) for the proportion of R responses, but “anecdotal evidence” for the alternative hypothesis (BF_10_ = 1.541) for the adjusted R score, suggesting the relationship between recognition performance and ‘Remember’ responses is inconclusive. Analysis of ERP data revealed a significant positive correlation between R/K effect magnitude and R/CR effect magnitude (*r* = 0.591, *p* = 0.006, BF_10_ = 9.325), with Bayes Factor analysis indicating substantial evidence for the alternative hypotheses. Despite the clear evidence that participants were recollecting, but replicating the findings of Experiment 1 and the SM task, there were no significant correlations between R/K effect magnitude (average of electrodes P5, P3, P1) and *Pr* (*r* = -0.183, *p* = 0.441, BF_10_ = 0.366, see **Figure 5C** (right) for a scatterplot depicting the relationship between R/K effect magnitude and *Pr*), *Br* (*r* = -0.182, *p* = 0.443, BF_10_ = 0.365), proportion of ‘Remember’ responses made (*r* = 0.129, *p* = 0.589, BF_10_ = 0.317), or adjusted ‘Remember’ responses (*r* = 0.357, *p* = 0.122, BF_10_ = 0.848). The Bayes Factor score for correlations with *Pr*, *Br*, and adjusted R were largely inconclusive, showing on the whole only “anecdotal evidence” for the null hypothesis, however, the Bayes Factor score for proportion of R show “substantial evidence” in support of the null hypothesis. Furthermore, there were no significant correlations between Remember/CR effect magnitude (average of electrodes P5, P3, P1) and *Pr* (*r* = 0.020, *p* = 0.933, BF_10_ = 0.278), *Br* (*r* = -0.256, *p* = 0.275, BF_10_ = 0.483), proportion of ‘Remember’ responses made (*r* = 0.022, *p* = 0.926, BF_10_ = 0.278), or adjusted ‘Remember’ responses (*r* = 0.147, *p* = 0.536, BF_10_ = 0.331). The Bayes Factor score indicate “substantial evidence” in support of the null hypothesis for R-CR correlations with *Pr*, proportion of R, and adjusted R, with only “anecdotal evidence” for the null hypothesis found for *Br*. A sensitivity analysis of a bivariate normal model correlation using G^∗^power 3.1 software (Faul et al., 2009) with a two tailed alpha value of 0.05 showed that a sample size of 20 with a power of 0.8 could detect an effect size of 0.579, with a correlation interval of ±0.444. A sample size of 20 would therefore only detect a very large effect.

A large number of participants (*n* = 33) were lost in the above analysis due to insufficient numbers of artifact-free ‘K’ trials. Sample size, and consequently power, could be increased for analyses not including K trials (R vs. CR) to 53 participants [excluding four participants due to low discrimination accuracy (*Pr* < 0.2) and six participants due to an insufficient number of artifact-free R trials (<16)].

Behavioral data is presented in **Table 2**, row C. Participants were successfully able to complete the task with mean discrimination accuracy above chance [*t*(52) = 21.05, *p* < 0.001]. Overall participants exhibited a conservative decision bias, and on average classified 67% (*SD* = 18%) of Hit trials as being ‘Remembered’, 25% (*SD* = 15%) as ‘Known,’ and 8% (*SD* = 8%) as ‘Guesses.’ Mean adjusted R = 53% (*SD* = 24%). ‘Guess’ responses were eliminated from any further analysis. There were no significant difference between the Hit and CR response times [*t*(52) = -1.84, 0.071].

**FIGURE 6:**
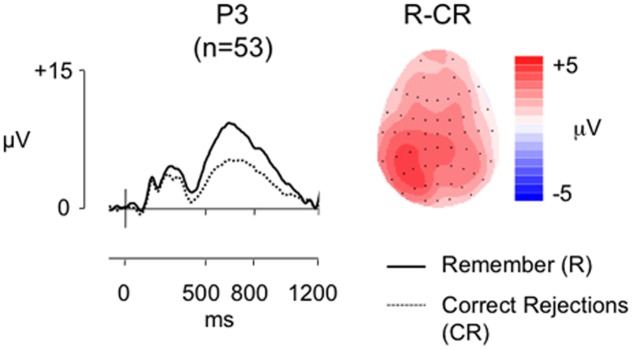
Grand-average ERP old/new effects from the RKG task (*n* = 53), at electrode P3, for ‘Remember’ (R) and CR responses, alongside a topographic map illustrating the clear left parietal distribution of the R-CR effect in this larger sample (scales as per **Figure 1**).

**Figure 6** shows grand average ERPs for the larger sample at electrode P3 for R and CR responses (mean number of R/CR trials = 40/63; minimum trials = 18/32; maximum = 88/86; mean percentage of trials rejected = 18%/24%), alongside a topographic map showing the old/new effect distributions between 500 and 800 ms post-stimulus. As expected there is a clear left parietal old/new effect in this larger sample. As in the smaller sample analysis we characterized the effects using ANOVA (**Table 4**, column 4). There was a significant main effect of condition all interactions with condition were also significant, clearly showing that R responses were more positive than CR responses, a difference greatest at parietal locations where it is greatest over left inferior and medial electrodes compared to a more widespread distribution across frontal locations.

In contrast to the smaller sample analysis the behavioral data revealed a significant positive correlation between the proportion of R responses and *Pr* (*r* = 0.433, *p* = 0.001), and between adjusted R and *Pr* (*r* = 0.657, *p* < 0.001), suggesting that in the larger sample as recognition performance improved the proportion of ‘Remember responses’ made increased. Bayes Factor analysis (BF_10_ = 27.828/BF_10_ = 177989.49, respectively) indicate “strong evidence” and “decisive evidence” for the alternative hypothesis, suggesting that those better at the recognition task experienced a sense of recollection more often. Despite the clear evidence that participants were recollecting, but replicating the findings of Experiment 1 and the SM task, there were no significant correlations between Remember/CR effect magnitude (average of electrodes P5, P3, P1) and *Pr* (*r* = -0.136, *p* = 0.333, BF_10_ = 0.271), *Br* (*r* = -0.108, *p* = 0.441, BF_10_ = 0.229), proportion of ‘Remember’ responses made (*r* = 0.016, *p* = 0.910, BF_10_ = 0.172) or adjusted R (*r* = 0.70, *p* = 0.616, BF_10_ = 0.194). The Bayes Factor score for all these correlations indicate “substantial evidence” in support of the null hypothesis. A sensitivity analysis of a bivariate normal model correlation using G^∗^power 3.1 software (Faul et al., 2009) with a two tailed alpha value of 0.05 showed that a sample size of 53 with a power of 0.8 could detect a medium to large effect size (Cohen, 1988) of 0.373, with a correlation interval of ±0271.

Experiment 2 re-examined the relationship between parietal activity and memory retrieval, using SM and Remember/Know tasks to assess recollection. Across the two tasks overall recognition rates were high (mean *Pr* > 0.5 in both tasks), as were recollection rates [64% Source Correct; 51% (*n* = 20) and 67% (*n* = 53) R responses]. Contrary to expectations, however, the left parietal effect simply did not vary systematically with behavioral estimates of recollection across participants. In the SM task and larger sample analysis of the RKG task recollection rates correlated significantly with overall recognition – suggesting that performance variability in Experiment 1 did indeed reflect changes in the rate of recollection across participants. Importantly, Experiment 2 also provided a clear replication of the ERP findings from Experiment 1: even when restricted to analysis of recollection based responses, left parietal old/new effect magnitude did not correlate significantly with behavioral measures of memory – either in discrimination accuracy, bias, or recollection rate. We cannot, of course, rule out the possibility that the measures we employ are, for unknown reasons, only sensitive to within (and not between) participant relationships. Regardless, it remains the case that these measures are used routinely to assess the neural correlates of recognition memory both within and between participants. Put simply, our findings demonstrate that across participants, neither typical objective nor subjective measures of recollection were systematically related to the magnitude of the left parietal old/new effect.

The preceding results are clear and consistent in themselves, raising questions about the reliability of the relationship between the left parietal effect and behavioral measures of memory retrieval. The results reported thus far have, however, relied exclusively on between participants analysis: this is in contrast to the within participant approach that has typically been employed in studies showing modulations of the left parietal effect as a function of memory (as outlined in the section “Introduction”). Thus, before considering the theoretical implications of our findings in detail, we present two further within participant analyses. First, we ask whether the left parietal old/new effect in our data is modulated in the way typically shown within the literature – that is, within participant, within task. To assess this, we reanalyzed the SM data to directly compare ERPs to Source Correct and Source Incorrect responses, with the expectation that the left parietal effect should be larger when source judgments were made correctly (as per Wilding and Rugg, 1996; Cruse and Wilding, 2009). Furthermore, we reanalyzed the RKG data to directly compare ERPs to ‘R’ and ‘K’ responses, with the expectation that the left parietal effect should be larger for ‘Remembered’ words (Düzel et al., 1997; Curran, 2004). This is important, because failure to demonstrate the typical modulation of the left parietal old/new effect would cast doubt on the generalizability of our overall pattern of results. Second, we ask whether the data reveal a relationship between parietal activity and memory performance when examined within participants, but across tasks. To assess this, we reanalyzed the SM and R/K/G data, to compare left parietal activity magnitude when participants performed two different memory tasks. Critically, this analysis provides insight into the stability of behavioral and neural measures of memory within individuals.

#### Within Participant Analysis

##### Is parietal activity modulated within participants?

As discussed in the section on ‘an objective measure of recollection,’ **Figures 4A,B** show grand average ERPs at electrode P3 for Hit Source Correct (HSC), Hit Source Incorrect (HSI), and CR responses (*n* = 61), alongside topographic maps showing old/new effect distributions between 500 and 800 ms post-stimulus. A repeated measures ANOVA comparing left parietal old/new effect magnitude (average of electrodes P5, P3, P1) across HSC, HSI, and CR groups confirmed the presence of significant differences in left parietal old/new activity between conditions [*F*(2,104) = 35.73, *p* < 0.001], with robust differences evident between HSC and CR responses [*t*(60) = 7.48, *p* < 0.001], HSI and CR responses [*t*(60) = 5.36, *p* < 0.001], and HSC and HSI responses [*t*(60) = 3.042, *p* = 0.003]. As can be seen in **Figure 4A**, when analyzed within participants, parietal activity is clearly modulated by SM accuracy, with a larger left parietal old/new effect present in ERPs for correct than incorrect source judgments.

As discussed in the section on ‘a subjective measure of recollection,’ **Figures 5A,B** show grand average ERPs at electrode P3 for R, K and CR responses (*n* = 20), alongside topographic maps showing the distribution of old/new effects between 500 and 800 ms post-stimulus. A repeated measures ANOVA comparing left parietal old/new effect magnitude (average of electrodes P5, P3, P1) across R, K, and CR responses confirms the presence of significant differences in left parietal old/new activity between conditions [*F*(2,33) = 9.72, *p* = 0.001], with strong differences evident between R and CR [*t*(19) = 4.93, *p* < 0.001], and R and K responses [*t*(19) = 2.80, *p* = 0.012]. No significant statistical difference was found between K and CR responses [*t*(19) = 0.98, *p* = 0.34]. As expected, analysis confirms that within participant, parietal activity is modulated by subjective experience of memory, with a larger left parietal old/new effect for words participants felt they ‘Remembered’ compared to words just ‘Known.’

##### How stable are parietal activity and behavioral indicators of memory across tasks?

Forty-nine participants met the inclusion criteria for both SM and RKG tasks. Participants were excluded for task non-completion (SM task = 1, RKG task = 3), low (*Pr* < 0.2) discrimination accuracy (SM task = 1, RKG task = 4), and insufficient number (<16) of artifact-free trials [SM task = 4, (HSC, HSI, or CR), RKG task = 5 (R or CR trials)] allowing comparisons of ERP and behavioral measures across tasks. Average ERPs were formed for each participant (SM task trials: Hit/CR mean = 100/115, respectively, minimum trials = 32/74, maximum trials = 108/142, mean trials rejected = 16%/21%; RKG task trials: R/CR mean = 40/63, respectively, minimum trials = 18/32, maximum trials = 88/86, mean trials rejected = 18%/24%). Mean recollection/CR effect magnitude was calculated for each task, and correlation analyses were run to test the relationship between neural measures across task, and between behavioral measures.

**FIGURE 7:**
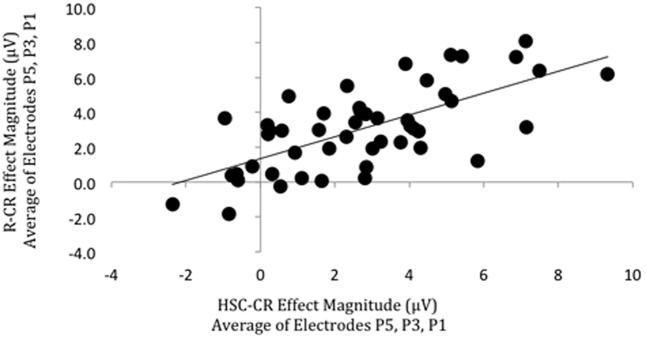
Scatterplot depicting the relationship between ‘Remember’/CR (vertical axis) and Hit Source Correct (HSC)/CR (horizontal axis) ERP effect magnitude in microvolts from 500 to 800 ms at left parietal electrodes (data averaged across electrodes P5, P3, P1). A significant correlation was found.

Participants in this subgroup had a mean Hit rate of 71% (14%)/73% (16%), False Alarm rate of 13% (11%)/18% (14%), *Pr* of 0.58 (0.15)/0.56 (0.19), *Br* of 0.31 (0.22)/0.40 (0.24), *Hit* RT of 1181 (282 ms)/1149 ms (243 ms), CR RT of 1200 ms (549 ms)/1212 (311), and percentage of ‘recollection’ (HSC/R) responses 64% (11%)/66% (18%) on the SM and RKG tasks, respectively (standard deviations given in brackets). There were positive significant correlations between behavioral memory measures [*Pr* (*r* = 0.655, *p* < 0.001, BF_10_ = 55942)*; Br* (*r* = 0.639, *p* < 0.001, BF_10_ = 24465), with Bayes Factor values indicating “decisive evidence” for the alternative hypothesis, however, no significant correlation was found for the proportion of recollection (as indexed by HSC or ‘Remember’ responses) trials (*r* = 0.246, *p* = 0.088, BF_10_ = 0.730)], with Bayes Factor scores inconclusive, showing only “anecdotal evidence” in support of the null hypothesis. Strong relationships were found between overall memory ERP measures (**Figure 7**), with a significant positive correlation in left parietal old/new effect magnitude (HSC-CR/R-CR, averaged across electrodes P5, P3, P1) across tasks (*r* = 0.677, *p* < 0.001, BF_10_ = 182075), with Bayes Factor values indicating “decisive evidence” for the alternative hypothesis in both cases. As per the behavioral analysis no significant correlation (*r* = 0.380, *p* = 0.098, BF_10_ = 0.994) between task recollection ERP measures (HSC-HSI/R-K) was found with the subset of 20 participants with enough ‘Know’ trials for analysis. The Bayes Factor score was inconclusive, showing only “anecdotal evidence” in support of the null hypothesis. A sensitivity analysis of a bivariate normal model correlation using G^∗^power 3.1 software (Faul et al., 2009) with a two tailed alpha value of 0.05 showed that a sample size of 49 with a power of 0.8 could detect a medium to large effect size (Cohen, 1988) of 0.387, with a correlation interval of ±0.282.

### Summary

Analysis of within participant data from Experiment 2 confirms that the left parietal effect modulates within participants in relation to objective SM accuracy (effect magnitude greater for Source Correct compared to Source Incorrect) and subjective experience of remembering (effect magnitude greater for ‘Remember’ than ‘Know’ responses). One interesting outcome to note is the clear difference in degree of response modulation across tasks, with left parietal effect magnitude modulated by each type of objective memory response (HSC > HSI > CR), whereas only R responses differed significantly from other responses in the subjective memory task (R > K = CR). This pattern of modulation is evident in the wider literature when comparing findings from studies using objective and those using subjective memory tasks, suggesting the two tasks are measuring slightly different aspects of recollection. Importantly, for current purposes, both tasks elicit the typical pattern of left parietal effect modulation evident in the literature (for SM see Wilding and Rugg, 1996; Cruse and Wilding, 2009, for R/K see Düzel et al., 1997; Curran, 2004), suggesting the absences of significant correlations across participants are not the result of unique task specific characteristics employed here. Confirmation that the tasks are not inherently different from those used in the wider field suggests the overall findings that behavioral and ERP measures of memory do not correlate across participants is likely to be generalizable, and not specific to the current study.

Comparisons of left parietal effect magnitude across SM and RKG tasks for overall memory (HSC-CR/R-CR) revealed significant positive correlations of left parietal effect magnitude across tasks, and significant correlations in behavioral measures of *Pr* and *Br*, but not the specific recollection contrast left parietal effect magnitude (HSC-HSI/R-K) or proportion of recollection trials. Theoretically, the positive correlation of left parietal effect magnitudes reflects consistent engagement of neural processing across tasks (whatever that processing supports cognitively), and the significant correlations in behavioral measures of *Pr* and *Br* suggests that memory performance across the two tasks is stable, with some participants performing better on both tasks than others. The inconsistency of the ‘recollection’ measures could be explained by the different nature of this measure in each task, which cannot be assumed to be directly comparable (an objective measure of recollection in the SM task and a subjective measure in the RKG task), or for the ERP measures may reflect the reduced power of the smaller sample.

**FIGURE 8:**
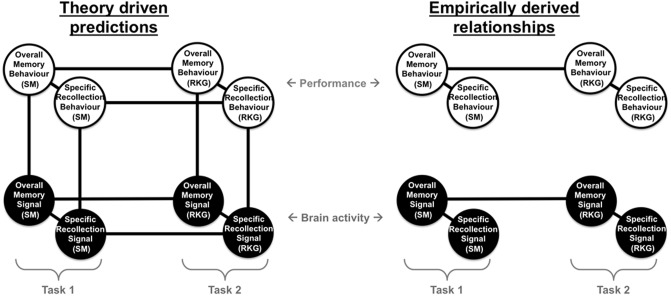
Summary diagram illustrating the theoretically driven predicted relationships (left) and empirically derived relationships found (right) between different behavioral measures of memory (behavioral measures of overall memory – *Pr* and specific recollection measures – Source Memory (SM) accuracy/Remember response rate), and neural measure of brain activity (neural signal of overall memory – magnitude of left parietal HSC-CR/R-CR ERP effect, and specific recollection measures – magnitude of left parietal HSC-HSI/R-K ERP effect), across two tasks capturing objective measures of recollection (SM) and subjective experiences of remembering (Remember/Know/Guess – RKG).

We highlight the overall pattern of findings in **Figure 8**, illustrating the relationship between behavioral and neural measures of memory. Based on theoretical assumptions we predicted (left) that there would be significant relationships between ERP and behavioral measures both within task with significant positive correlations between behavioral measures of overall memory (i.e., *Pr*), behavioral measures of specific recollection (i.e., SM accuracy/proportion of ‘Remember’ responses), neural signal of overall memory (i.e., left parietal effect magnitude for HSC-CR/R-CR), and neural signal of specific recollection (i.e., left parietal effect magnitude for HSC-HSI/R-K), and between task (with significant positive correlations between the two tasks for behavioral, and ERP measures). However, the results presented here indicate that the empirically derived relationships (right) are more complex, with significant relationships found within tasks, within-measures (significant positive correlations found between behavioral measures of overall memory and specific recollection, and between ERP measures of overall memory and specific recollection), but not between measures (no correlation between behavioral and ERP measures of either overall memory or specific recollection). Furthermore, significant relationships were found across tasks for ERP measures of overall memory [significant positive correlations between left parietal effect magnitudes (HSC-CR/R-CR), on task one and task two], but not ERP measures of specific recollection (HSC-HSI/R-K). Similarly significant relationships for behavioral measures were only found for overall memory (significant positive correlations between *Pr* scores between tasks 1 and 2), but not specific recollection measures (no correlation between SM accuracy/R rate between task one and task two). Bottom line, our data demonstrates that, at a population level in healthy young adults, between participant variation in the magnitude of the left parietal old/new effect does not correlate with recollection.

## General Discussion

Event-Related Potential studies of recognition memory have consistently shown increases in activity over left parietal electrodes for old items compared to new items during episodic retrieval. There is a clear view within the literature that this parietal activity provides an index of episodic memory retrieval. In the present paper we tested this principle across a large group of participants, examining the relationship between memory and the left parietal ERP old/new effect. As predicted, within participants, the magnitude of the left parietal effect increased in conditions associated with recollection – larger old/new effects were seen for R than K, and Source Correct than Source Incorrect ERPs. By contrast, to our surprise, but replicated over two experiments, population-level analyses revealed that variability in the magnitude of parietal activity does not correlate with variability in memory performance. The implications of these findings are clear: at least where data from healthy young adults are involved, the left parietal effect cannot be used to index changes in episodic recollection between participants, and comparisons between groups must be made with great care.

### The Left Parietal Effect Does Not Correlate with Recollection

Before considering the functional implications of the present findings, we first briefly highlight the key results and discuss potential limitations. Experiment 1 employed a simple item recognition task, revealing that ERP measures of retrieval were not related to behavioral performance, either in correlational analysis across the entire sample, or in targeted comparisons between groups of good and bad remembers. As a result, in Experiment 2 we replicated the findings using two complementary but distinct operational definitions of recollection – providing both objective (SM) and subjective (Remember/Know/Guess) measures – allowing us to more carefully target the putative retrieval process indexed by parietal activity. These two tasks capture different types of recollection, with the SM task capturing criterial recollection and the RKG task able to capture a broader array of recollection experiences. In this context the convergence of findings is particularly striking: across both objective and subjective measures of recollection, between participants, neural measures of episodic retrieval success (i.e., left parietal old/new effects) were not related to behavioral performance.

Although the present findings are unexpected, the use of large samples and replication adds considerably to the likely validity and generalizability of the results (Button et al., 2013). To be clear, the lack of correlation between ERP and behavioral measures of memory across individuals cannot be readily explained by poor statistical power. For example, Experiment 1 involved an unusually large sample of 122 participants. Moreover, sensitivity analysis indicated that the design provided sufficient power to detect a small to medium sized correlation between behavioral performance and the magnitude of the left parietal effect. Put another way, within participants the left parietal old/new effect was highly robust, yet there was no sign of a correlation between neural activity and performance across participants. In this context, the present results could be taken as evidence that even larger groups of participants are required to find population level between-participant correlations. To our minds, this would render the left parietal effect to be unusable in the context of traditional between participant studies (where the *N* is far lower). Regardless, in practice both behavioral performance and ERP measures exhibited a good degree of variability across participants, such that our dataset would have been well able to reveal a correlation if one had been present. Overall, the present data suggest that at a population level the left parietal effect simply does not correlate with behavioral measures of the rate of recollection.

One way of characterizing our findings is that they reveal a mismatch between behavioral and neural measures of memory. It is, of course, impossible to entirely rule out the objection that the results may reflect little more than the paucity of available behavioral measures. Source accuracy, R responses, reaction times, proportion of Hit responses, proportion of CR responses, *Pr* and *Br* may not be sufficient to characterize the key differences in memory across individuals. Furthermore, the absence of a ‘Guess’ response option in Experiment 1 and in the source judgment task of Experiment 2 may reduce the likelihood of seeing a correlation in either of these two tasks, with ‘Guess’ responses diluting any effects. However, ‘Guess’ responses were removed from analysis of the RKG task and there was no evidence of a correlation between the magnitude of the left parietal effect and behavioral performance. Moreover, the metrics we employed are amongst the most common and well-understood measures of episodic memory, and the absence of a significant relationship between behavioral and ERP measures of memory when using these primary methods cannot simply be ignored. In addition, one aspect of the findings serves to highlight that the disparity is not simply due to the use of poor behavioral measures. By examining good and bad performers we isolated clear behavioral differences that, in principle, must reflect variability in how much participants remembered – yet the magnitude of the left parietal effect did not differ between these groups. This finding reinforces the view that the left parietal old/new ERP effect does not index variability in recollection across participants.

**FIGURE 9:**
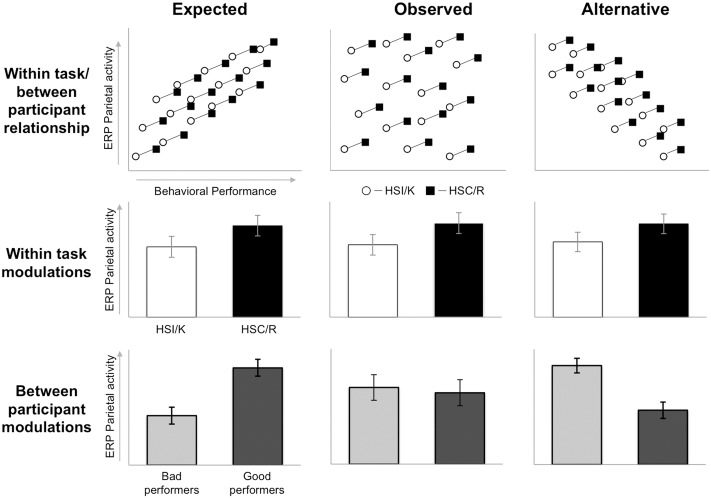
Three hypothetical datasets: ‘expected’ (left), ‘observed’ (center), and ‘alternative’ (right), examining the relationship between left parietal old/new ERP effects (vertical axis) and memory retrieval (horizontal axis), considering within task between participants patterns (top), modulations within task (middle), and modulations between participants (bottom). Black squares/bars represent trials with recollection (Source Correct and ‘Remember’ responses) and white circles/bars represent trials without recollection (Source Incorrect and ‘Know’ responses). Scatterplot lines connecting squares and circles identify data from the same participant. Light gray bars represent bad performers and dark gray bars represent good performers. Each dataset shows the same within task modulation (trials with greater recollection exhibit larger left parietal old/new effects than those without), but different patterns of between participant modulations.

An important feature of the current findings is that the data clearly replicates previously documented within participant left parietal effect modulations. That is, larger left parietal old/new effects were present for Source Correct than Incorrect responses, and for ‘Remember’ than ‘Know’ responses – the very pattern that originally motivated the view that the left parietal effect might *index* retrieval. To be clear, the same neural measures simultaneously exhibit within participant within task modulations, and no between participants relationship with memory. At first pass these results appear contradictory: can a single neural response show both of these patterns? The fact that both patterns can occur at the same time, for a single signal, is illustrated in **Figure 9**, which outlines three hypothetical sets of data. The left column illustrates our original anticipated pattern of results: increases in the magnitude of the left parietal effect both within and across participants. This data pattern would lead to both within task modulations (middle) and differences between good and bad performers (bottom), where the magnitude of the left parietal effect is greater for good performers. The center column illustrates the kind of pattern reported here: increases in the left parietal effect are associated with increases in memory within participant, within task (middle), but not across participants (bottom), where there is no difference in left parietal effect magnitude across participants who are good vs. bad at remembering. Finally, for completeness, the right column illustrates a third possible result: the size of the left parietal effect simultaneously increases with recollection within each participant, within task (middle), yet decreases with retrieval across participants (bottom), leading to a larger left parietal effect for those who are bad at remembering. This later pattern has not, to our knowledge, been reported in the ERP literature, and would be an illustration of Simpson’s Paradox (Kievit et al., 2013).

**Figure 9** illustrates how the current data reconcile existing findings within the ERP literature – explaining why researchers have repeatedly failed to find group differences between good and bad performers (e.g., Van Petten et al., 2002; Wolk et al., 2009). Rather than reflecting a failure to find a difference (as is often claimed, e.g., on the basis of lack of power), such studies have in fact accurately reported no group level difference in parietal activity. By extension, the absence of across participant relationships can also explain the failure to find left parietal magnitude differences between groups, such as older and young participants (Mark and Rugg, 1998), despite differences in memory performance. More broadly, whilst the demonstration of different patterns within and between participants is surprising when viewed within the local field, similar patterns have been demonstrated in a range of other contexts. For example, Boy and Sumner (2014) examined behavioral measures of priming, revealing a pattern of strong within participant modulations but no between participant effects. Similarly, Guerra et al. (2016) report differential within participant and between participant relationships between Heart Rate variability and P300 ERP amplitude. Taken together, these studies suggest that examining population level data is important for fully understanding the functional utility of any given measure, and highlight the danger of extending conclusions drawn from within participant paradigm designs to between participant comparisons.

Within the wider literature it is recognized that any pattern of within participant variability is compatible with any pattern of between participant variability (cf. Borsboom et al., 2009, as illustrated in **Figure 9**). Distinctly different within and between participant patterns can occur, for example, when variance between individuals arises from different sources than variance within individuals (as has been demonstrated for complex psychological traits such as personality and IQ). However, even counter-intuitive patterns such as the opposite within and between effects shown in **Figure 9** (right) do not *require* that sources of between participant variance are entirely different from the sources of within participant variance. Even apparently simple variables can exhibit paradoxical patterns when viewed within vs. between participants. For example, consider the relationship between amount of sleep and use of sleeping tablets. Viewed across individuals we would expect a negative relationship (decreased amounts of sleep being associated with increased use of sleeping tablets), but viewed within individuals we would expect the opposite pattern to hold (increased use of sleeping tablets associated with increased amounts of sleep). In this example sleeping tablets are related both positively and negatively with amount of sleep, depending on whether we are looking within or between participants. By analogy, our ERP findings demonstrate that the left parietal old/new effect is related to episodic recollection, but only within and not between participants – this pattern appears likely to reflect a real property of the data (cf. Borsboom et al., 2009), rather than simply a failure to detect a relationship in the between participant case.

### The Left Parietal Effect Is Stable within Individuals

A novel and potentially important aspect of the current data is the presence of significant within participant across task correlations between left parietal effect magnitudes, suggesting that the ERP measure is stable within individuals. One potential explanation for the stability of ERP measures is that variability in left parietal effect magnitude across participants may reflect genuine differences in neuroanatomy. For example, individual differences very likely exist in the depth and orientation of the underlying neural generators, which would produce variability in the magnitude of the left parietal effect across individuals, but would be stable within individuals. To be clear, the generator of the effect may be located in a manner that results in larger or smaller old/new effects, regardless of the level of remembering that occurs. Equally, one could also argue that variability in factors such as skull thickness, head size and electrode impedance could all impact on how much neural activity can be measured at the scalp – again independent of how much recollection is occurring.

To the extent that the measurement of neural activity is affected by such factors, one could argue that the absence of a correlation with memory performance simply reflects the limitation of the psychometric properties of behavioral measures, or the noisiness of electrophysiological measures, which may suppress any inter-individual correlations, rather than the absence of a relationship *per se*. Whilst our data cannot rule out neuroanatomical variability as responsible for the lack of a correlation with performance, we are able to definitively rule out head size as a factor. ERP procedures require head measurement, and the use of different sized caps – allowing us to examine the magnitude of the old/new effect as a function of cap size. Notwithstanding the fact that head size is itself a relatively weak measure of neuroanatomical variability, analysis of the data contingent on head size demonstrates that variability in head size does not account for the variability in old/new effect size across participants.

More importantly, our view is that even if neuroanatomical variability or wider measurement limitations (behavioral or electrophysiological) are responsible for the lack of correlation between ERP and behavioral measures, the absence of a correlation with performance within participants, across task, still strongly suggests that the left parietal effect does not reliably predict memory performance. To be clear, the present data, suggests that knowing the size of an individual participant’s left parietal effect, does not provide any indication as to whether the individual has remembered or not. Equally, our findings suggest that if one were to compare the magnitude of the left parietal effect across two groups of participants, the relative size of the effect does not provide any indication as to which participants had remembered better or worse. As we outlined in the introduction, many ERP studies rely on exactly this kind of comparison, including for the important investigations of aging, development and disease: the present findings strongly suggest that the left parietal effect may not be effective for these purposes, particularly where comparisons with healthy young adult data are involved. Again, this finding resonates with broader concerns about the relationship between within and between participant variance in psychological processes. Notably, Borsboom et al. (2009, p. 73) argue that within and between participant findings can only converge when “inter-individual differences found in correlational research are exclusively the result of the intra-individual processes studied in the corresponding experimental research” – circumstances that they believe to be “highly limited.”

Although the present findings provide an important constraint on the interpretation and use of the left parietal effect as a neural correlate of recollection, it is important to acknowledge that they do not rule out its use entirely. First, reliable within task, within participant modulations remain – questions that rely solely on this kind of comparison appear to be entirely valid. Second, the lack of correlation with performance is a useful caution against the comparison of old/new magnitudes across groups as a means of assessing variability in recollection. To be clear, however, we do not believe that group ERP studies (e.g., involving aging, developmental or disordered populations) are inherently flawed. For example, the present findings do not rule out consideration of differences in the time-course or distribution of effects, which can be used to infer changes in the timing of processing (cf. Mark and Rugg, 1998) and qualitative differences in pattern of processes engaged (cf. MacLeod and Donaldson, 2014), respectively. Third, the present findings relate to recognition memory for word stimuli and therefore do not allow strong conclusions to be drawn about the relationship between the old/new effect and behavioral performance when examining other stimuli, such as pictures or faces. Fourth, given the stability of the left parietal effect within individuals, it remains possible that it could still provide a useful index of memory within each individual – for example to track changes in memory over time, within task, within participant. To our knowledge no existing study has examined the left parietal ERP old/new effect in this way, at an individual level, making this an important avenue for future research. Finally, it is important to note that whilst the variability in left parietal effect magnitude did not predict variability in memory performance in this study, the findings do not imply that this is always the case. It remains possible that within other populations, in whom variance in memory performance derives from different causes (e.g., neurological patients), it may be possible to predict performance from effect magnitude. Furthermore it may be possible that finer grained contrasts which allow mean participant effects, such as neural generator configuration or individual head conductance, to be effectively subtracted out, may in future reveal valid between participant associations. Nonetheless, the lack of detectable associations within a sample of healthy young adults, using a variety of basic standard recognition tasks, provides caution for interpreting between participant variance.

### The Left Parietal Effect May Reflect the Quality Rather than Quantity of Recollection

One interpretation of our findings is that existing measures of episodic memory simply do not adequately capture the nature of cognitive processing involved. There are many alternative tasks that could be employed to assess episodic recollection, including cued and free recall, as well as meta-memory judgments of decision confidence or vividness. To our minds, however, there is no obvious theoretical rationale for arguing that the use of these other measures would remove the difference between within and across participant effects observed in the present study. One important issue for future studies is to examine the possibility that other processes (i.e., tonic state related activity, variation in attention, or differences in encoding or post-retrieval processing) are critical in determining the rate of recollection, complicating the assessment of between participant variability in memory. Nonetheless, given the current data we consider it to be more plausible that the traditional functional characterization of the left parietal effect is problematic. One potential alternative functional account of the parietal effect is that it reflects variability in the quality or richness of retrieval (rather than the amount or quantity of retrieval). Here we ask whether this alternative account could reconcile the within and between patterns of data observed in the present study.

Evidence linking parietal activity with the quality of memory can be found in studies designed to reconcile patient data with findings from neuroimaging. For example, Berryhill et al. (2007) suggest that while patients suffering bilateral parietal lobe damage are not amnesic, they do suffer subtle episodic memory deficits. Berryhill et al. (2007) found that although patients performed as well as controls when asked specific questions, they provided less detail about their memories during free recall. Similarly, Simons et al. (2010) argue that patients with bilateral parietal lobe damage show reduced confidence in their ability to recollect source information – despite actually performing as well as matched control participants. These findings therefore suggest that whilst patients with parietal lobe damage do not suffer from amnesia, they do show some evidence of episodic memory impairment, particularly in relation to subjective measures of memory and ‘richness’ of the experience of retrieving. Most importantly for present purposes, evidence from Vilberg et al. (2006) showed that the left parietal effect was sensitive to the amount of information recollected, with items with full recollection showing greater amplitude than items with only parietal recollection. In addition, recent evidence from Murray et al. (2015) suggests that within participant variability in the magnitude of the left parietal old/new effect is specifically sensitive to the quality of recollection – as measured by the precision of SM responses. In this case, comparisons were made within participants: even when the rate of recollection was matched across conditions the magnitude of the left parietal effect correlated with the quality of recollection responses (the precision of remembered spatial location information).

If the present findings are viewed in terms of the ‘richness’ of retrieval, then the lack of correlation between the left parietal effect and task performance might be seen as less surprising. According to this view, behavioral measures of task success do not correlate with neural measures because they do not provide information about the richness of the retrieval experience. One can easily imagine that individuals differ in terms of how vivid or rich their memories are, and that without appropriately scaling for these differences, no between participant correlations will be found. Although superficially attractive, at present we see relatively little evidential reason to support this alternative phenomenological account. First, in the present study we employed the most widely used measure of the subjective experience of recollection (R/K/G task, which provides a measure of the quality of memory experience) – yet no across participant correlation was found between parietal activity and the proportion of ‘Remember’ responses made. Second, the philosophical challenge associated with assessing qualia seems unlikely to be overcome easily. Nonetheless, taken together, the present findings and those of Vilberg et al. (2006), and Murray et al. (2015) suggest the need for better understanding of the typical within task modulation of the left parietal effect. Future research is needed to target variation in the quality of memory experience (e.g., vividness, distinctiveness, etc.), rather than just the likelihood that recollection has occurred.

Although the current data could in principle be explained in terms of the quality and/or richness of experience, a number of other memory models struggle to readily explain our current findings. One recent proposal has been to characterize parietal contributions to memory as reflecting the operation of an ‘episodic buffer’ (Baddeley, 2000; Vilberg and Rugg, 2008) that acts as an interface between working and long-term memory, temporarily storing and integrating information from a variety of systems into an episodic representation. To our reading, the episodic buffer account predicts that left parietal activity would be seen in all participants, because all participants were remembering and must therefore have held remembered information in mind. As **Figure 2** highlights, however, not all participants exhibited positive going parietal old/new effects – despite performing the task at well above chance levels. A similar problem exists for ‘attention to memory’ accounts (Ciaramelli et al., 2008) that view parietal activity as reflecting the direction and maintenance of attention to internal mnemonic representations. To our reading, it is not clear how this kind of model can account for participants who are successfully able to retrieve memories (and therefore were presumably attending to those memories) but who nonetheless exhibit small, or no, parietal old/new effects. Whilst both of these proposals have merit as an explanation of parietal functioning *per se*, it is unclear how they can accommodate the population level variability reported here. One alternative possibility is that recollection is simply an epiphenomenon, with changes in the magnitude of the effect occurring when control has been exerted over recollected information. This view stems from studies examining correlations between working memory capacity and SM based target detection (Elward and Wilding, 2010; Elward et al., 2013; see also Sprondel et al., 2012). Together, these studies suggest that the left-parietal effect may reflect processes supporting the active maintenance of target information in working memory – rather than reflecting retrieval *per se*. Again, however, it remains unclear how this kind of account can explain the absence of old/new effects seen in many participants here. Regardless of which account proves correct, functional accounts of the left parietal old/new effect must be able to explain why different patterns of data occur within and between participants.

## Conclusion

The present findings provide new insight into the functional utility of the left parietal old/new ERP effect during episodic memory. Despite evidence of within participant and within task left parietal effect modulations with recollection, in our sample of health young adults behavioral performance could not be inferred from the magnitude of the left parietal effect across participants. We argue that the observed pattern of effects is likely to be a real feature of ERP memory data, rather than simply an artifact of poor measurement, and is consistent with wider demonstrations of conflicting inter- and intra- individual differences. Our data suggests that the relationship between the left parietal effect and recollection is more complex than previously thought: put simply, variation in the magnitude of the left parietal old/new ERP effect does not always reliably predict variation in episodic recollection between participants. As a result, functional interpretations of between-group variance in the magnitude of the left parietal effect must be made with caution.

## Author Contributions

CM contributed to the conception and design of the work, data collection/analysis/interpretation, and drafting/revising the manuscript. DD contributed to the conception and design of the work, data interpretation, and drafting/revising the manuscript. All authors approved the work for publication, and agree to be accountable for all aspects of the work.

## Conflict of Interest Statement

The authors declare that the research was conducted in the absence of any commercial or financial relationships that could be construed as a potential conflict of interest.

## Footnotes

1 http://www.neuroscan.com
